# HSV1 infection elicits distinct immune responses in microglia and macrophages

**DOI:** 10.1002/alz.092417

**Published:** 2025-01-03

**Authors:** Chitra Mohinder Singh Singal, Elizabeth M Bradshaw, Wassim Elyaman

**Affiliations:** ^1^ Columbia University Irving Medical Centre, New York, NY USA; ^2^ Columbia University Irving Medical Center, New York, NY USA; ^3^ Department of Neurology, Columbia University, New York, NY USA

## Abstract

**Background:**

Alzheimer’s disease (AD) is the most common form of dementia. The neurotropic virus herpes simplex virus 1 (HSV1) has been linked to the pathogenesis of AD. While ∼65% of the US population is infected with HSV1, in the majority of cases the virus is dormant and infected individuals are asymptomatic. HSV1 infects immune cells such as macrophages and microglia, essential components of the innate immune response that are involved in neuroinflammation and neurodegeneration in AD.

**Method:**

Monocytes were isolated from PBMCs of healthy donors and differentiated into macrophages and monocyte‐derived microglia‐like cells (MDMi) in the presence of stimulatory cytokines for 6 days and 10 days respectively. The differentiated cells were infected with HSV1 and the cytokine profiles were measured by the LegendPlex assay. Additionally, fluorescently‐labeled Abeta1‐42 uptake was analyzed after 24 hours of infection.

**Result:**

There was a significant increase in several inflammatory cytokines including IL‐4, IL‐6, TNFα, and IP‐10 in macrophages, whereas there was no significant change in MDMi after 4 or 24 hours of infection. Interestingly, Abeta1‐42 uptake in HSV1‐infected macrophages was decreased compared to their controls, whereas in HSV1‐infected MDMi, there was an enhancement of Abeta1‐42 uptake.

**Conclusion:**

Macrophages exhibit an enhanced inflammatory response in contrast to microglia upon HSV1 infection and an opposite reaction in Abeta1‐42 uptake. Our findings indicate distinct responses of these two cell types to HSV1 infection, providing novel insight into our understanding of their unique tissue‐specific roles.

